# The TNF Receptors p55 and p75 Mediate Chemotaxis of PMN Induced by TNFα and a TNFα 36–62 Peptide

**DOI:** 10.1155/S0962935194000487

**Published:** 1994

**Authors:** Ø. Rekdal, Z. Konopski, J. S. Svendsen, J.-O. Winberg, T. Espevik, B. Østerud

**Affiliations:** 1 Institute of Medical Biology University of Tromsø Tromsø 9037 Norway; 2 Institute of Mathematical and Physical Sciences University of Tromsø Tromsø 9037 Norway; 3 Polar Institute of Medical Genetics University of Tromsø Tromsø 9037 Norway; 4 Institute of Cancer Research University of Trondheim Trondheim N-7006 Norway

## Abstract

The present study was performed to examine whether residues 36–62 of TNFα contain the chemotactic domain of TNFα, and whether the p55 and p75 TNF receptors are involved in TNFα induced chemotaxis. The chemotactic effect of TNFα on PMN was inhibited by the mAbs Hrt-7b and Utr-1, against the p55 and p75 TNF receptors, respectively. Both receptors may therefore be required for mediating the chemotactic effect of TNFcz. The synthetic TNFα 36–62, similar to TNFα, had chemotactic effects on both PMN and monocytes. The chemotactic activity of the TNFα 36–62 peptide on PMN, was inhibited by Htr-7b, Utr-1 and soluble p55 receptor, which shows that the peptide possessed the ability to induce chemotaxis through the TNF receptors. In contrast to TNFα, the peptide did not show a cytotoxic activity against WEHI 164 flbrosarcoma cells. It is suggested that different domains of the TNFα molecule induce distinct biological effects.


Mediators of Inflammation 3, 347–352 (1994)

### The TNF receptors p55 and p75 mediate chemotaxis of PMN induced by TNFα and a TNFα 36–62 peptide

Ø. Rekdal,[1,CA] Z. Konopski,[1] J. S. Svendsen,[2] J.-O. Winberg,[3] T. Espevik[4] and B. Østerud[1]

[1]Institute of Medical Biology, [2]Institute of Mathematical and Physical Sciences and [3]Polar Institute of Medical Genetics, University of Tromsø, 9037 Tromsø, and [4]Institute of Cancer Research, University of Trondheim, N-7006 Trondheim, Norway

[CA] Corresponding Author

**The present study was performed to examine whether residues 36–62 of TNFα contain the chemotactic domain of TNFα, and whether the p55 and p75 TNF receptors are involved in TNFα induced chemotaxis. The chemotactic effect of TNFα on PMN was inhibited by the mAbs Hrt-7b and Utr-1, against the p55 and p75 TNF receptors, respectively. Both receptors may therefore be required for mediating the chemotactic effect of TNFα. The synthetic TNFα 36–62, similar to TNFα, had chemotactic effects on both PMN and monocytes. The chemotactic activity of the TNFα 36–62 peptide on PMN, was inhibited by Htr-7b, Utr-1 and soluble p55 receptor, which shows that the peptide possessed the ability to induce chemotaxis through the TNF receptors. In contrast to TNFα, the peptide did not show a cytotoxic activity against WEHI 164 fibrosarcoma cells. It is suggested that different domains of the TNFα molecule induce distinct biological effects.**

**Key words:** Chemotaxis, Molecular modelling, Synthetic peptide, TNFα, TNF receptors

### Introduction

Circulating polymorphonuclear cells (PMN) and monocytes are activated by chemotactic factors for recruitment to sites of inflammation.[1] The pleiotropic cytokine, tumour necrosis factor-α (TNFα), has been reported to be chemotactic, as it induces directional locomotion of PMN and monocytes *in vitro*.[2–5] Furthermore, *in vivo* studies show that TNFα plays a crucial role in the recruitment of neutrophils at an early stage, and monocytes at a later stage of immune complex-induced inflammatory reactions.[6,7] Many biological effects induced by TNFα[8–11] have been shown to involve binding to the 55 kDa (p55) and the 75 kDa (p75) TNF receptors, which are expressed on almost all cell types.[10] However, the involvement of these receptors in the TNFα induced chemotaxis has not been studied.

An interesting approach for the study of distinct TNFα activities is the use of TNFα peptides. Different TNFα peptides have recently been reported to induce distinct TNFα effects,[12–14] and inhibit binding of TNFα to the TNF receptors.[13] The authors have performed molecular dynamic calculations[15] combined with studies on the three-dimensional structure of TNFα[16] in order to design TNFα peptides which could interact with TNF receptors, and induce TNFα effects. It was found that a peptide including residues 36–62 had conformational properties which could be related to the corresponding parent molecule. This sequence is also one of the most homologous domains between TNFα and TNFβ, which both bind to the TNF receptors.[16] In the present study, this peptide was investigated for two crucial TNFα effects, chemotaxis and cytotoxicity. The involvement of p55 an p75 TNF receptors in the chemotactic response of TNFα and TNFα 36–62, was also studied.

### Materials and Methods

*Molecular modelling:* The molecular modelling studies of TNFα peptides were performed using the Molecular Simulation Inc. Quanta 3.2/CHARMm 21.2 program package, on a Silicon Graphics Personal IRIS 4D/30 EG (USA). The peptide atom coordinates were obtained from the TNFα X-ray structure (pdbl tnf), and peptide candidates were minimized by molecular mechanics using 2000 steps of adopted basis set Newton–Raphson minimization, before the minimized structures were subjected to molecular dynamic calculations with a total simulation of 250 ps at 300 K.

*Synthesis of TNFα peptides:* TNFα peptides were synthesized as described previously,[17] using Fmoc chemistry on a semi-automatic peptide synthesizer (Milligen, Model 9020). The peptides were purified and analysed using reverse-phase HPLC, and FIB–MS on a VG Tribid MS instrument (VG Analytical, Manchester, UK).

© 1994 Rapid Communications of Oxford Ltd

*Materials:* Human recombinant TNFα (Hr TNFα), with a specific activity of $1.0 \times 10^8$ U/mg, was purchased from Boeringer (Mannheim, Germany). The generation of the mAbs Utr-1 an Htr-7b, specific for p75 and p55 respectively, is described elsewhere,[18] and soluble p55 was kindly provided by Dr Hansruedi Loetscher, Hoffman–La Roche (Basel, Switzerland). Anti-IL-8 and anti-MCP-1 were purchased from British Biotechnology (UK). Formylmethionyl-leucyl phenylalanine (FMLP) was purchased from Sigma Chemical Co. (St Louis, MO). Endospecy from Seikagaku Co. (Tokyo, Japan) was used to check endotoxin contamination.

*PMN isolation:* Polymorphonuclear cells (PMN) were isolated as follows: 2 ml of freshly drawn heparinized blood (10 U/ml) from healthy adults was applied on top of a bilayer consisting of 3 ml polymorphoprep and 3 ml lymphoprep in polycarbonate tubes (Nycomed Pharma AS, Norway). After centrifugation at 530 × **g** for 20 min, the PMN band in the polymorphoprep layer was isolated. The cells were washed once with ice cold sterile 0.15 M NaCl, and centrifuged at 185 × **g** for 10 min. Contaminating erythrocytes in the PMN band were lysed with ice cold 0.2% NaCl for 90 s. The cells were resuspended at $10^6$/ml in ice cold RPMI-1640 and used immediately. The PMN preparation contained at least 95% neutrophils.

*Monocyte isolation:* The monocyte band was isolated using the method previously described by Bøyum.[19] In brief, mononuclear cells (PMBC) from healthy adults, from either freshly drawn heparinized blood or buffy coats (10 U/ml), were centrifuged on lymphoprep, isolated and washed with 0.15 M NaCl. The PMBC, resuspended at $10^6$/ml in RPMI-1640 were used directly for chemotaxis studies.

*Assay for chemotaxis:* TNFα and TNFα peptides were tested for chemotactic activity on PMN and PBMC. Chemotactic activity was assayed in a 48-well microchemotaxis chamber (Neuro Probe Inc. Cabin John, MD, USA), as described previously.[20] In brief, the upper wells were filled with 50 μl of cells, and 25 μl of the compounds tested for chemotaxis were filled in the bottom wells. For checkerboard analysis, the stimulants were also placed in the upper wells. In the pretreatment studies, the anti-TNF receptor antibodies, or other antibodies were added to the cells for 10 min at 4°C, before they were placed in the upper wells. The soluble p55 was mixed with 0.5 μM TNFα 36–62 in a 1:1 molar ratio at 20°C for 10 min, before addition to the lower wells. A polycarbonate–polyvinyl pyrrolidone (PVP) filter with 5 μm pore size was used in the PBMC chemotaxis assay, while a PVP-free polycarbonate filter, with the same pore size, was used for the PMN chemotaxis assay. Chemotaxis chamber assemblies were incubated at 37°C in humidified 95% air and 5% $CO_2$ for 3 h in assays with PBMC and 40 min in assays with PMN. Then the filters were removed, fixed in 2.5% glutaraldehyde (Merck, Damstad, Germany), and stained with Giemsa (Sigma, Cleveland, USA) for 30 min. Cells that had migrated through to the bottom of the filter were counted in 6–10 high-power fields (HPF) (× 60 or 100 objective). Chemotactic bioactivity was expressed as the mean number of cells per HPF. Variations in response to the tested agents, were dependent on the blood donor.

*Assay for cytotoxicity:* Cytotoxicity of TNFα 36–62 was tested using the fibrosarcoma cell line WEHI 164 clone 13, as described by Espevik *et al.*[21] Cell viability in the assay was measured colorimetrically, by using tetrazolium salt (MTT), as described by Mosmann *et al.*[22]

### Results

*Molecular modelling of TNFα 36–62 peptide:* Starting from a minimized X-ray structure, the conformational properties of TNFα 36–62 in a vacuum environment were calculated using molecular dynamics. The results from the calculations suggested that the peptide would possess a partially conserved tertiary structure similar to the conformation in the minimized crystal structure of TNFα (Fig. 1). Parts of β-strands from each of the two β-sheets in the TNFα monomer are included in TNFα 36–62 (Figs. 1A and 1B). The β-strands in TNFα 36–62 were stabilized by hydrogen bonds, but some of these were different from the corresponding hydrogen bonds observed in the TNFα structure. A β-strand interruption in TNFα could also be recognized in the peptide. The two loops (38–41 and 50–54) located at the base of TNFα, and important residues surrounding a shallow depression which are suggested to be involved in receptor binding,[16] were all exposed in a similar manner in the peptide as in TNFα. The distance between the carbon in the C-terminal carboxyl and the nitrogen in the N-terminal amino group in TNFα 36–62 was only 2.89 Å compared to 18.19 Å in TNFα, indicating an attraction between the oppositely charged C- and N-terminals. The effect of this electrostatic attraction is, however, expected to be of much less importance in aqueous environment.

*Chemotactic effects of TNFα and TNFα 36–62 peptide on PMN:* TNFα and the TNFα 36–62 peptide have been tested for the ability to attract PMN. The experiments were repeated at least three times, and similar results were obtained despite donor variations. Both TNFα and TNFα 36–62 showed a dose dependent chemotactic effect on PMN. Migration of approximately 120 cells was achieved with either 2 nM TNFα or 10 μM TNFα 36–62 (Fig. 2). That a higher concen-

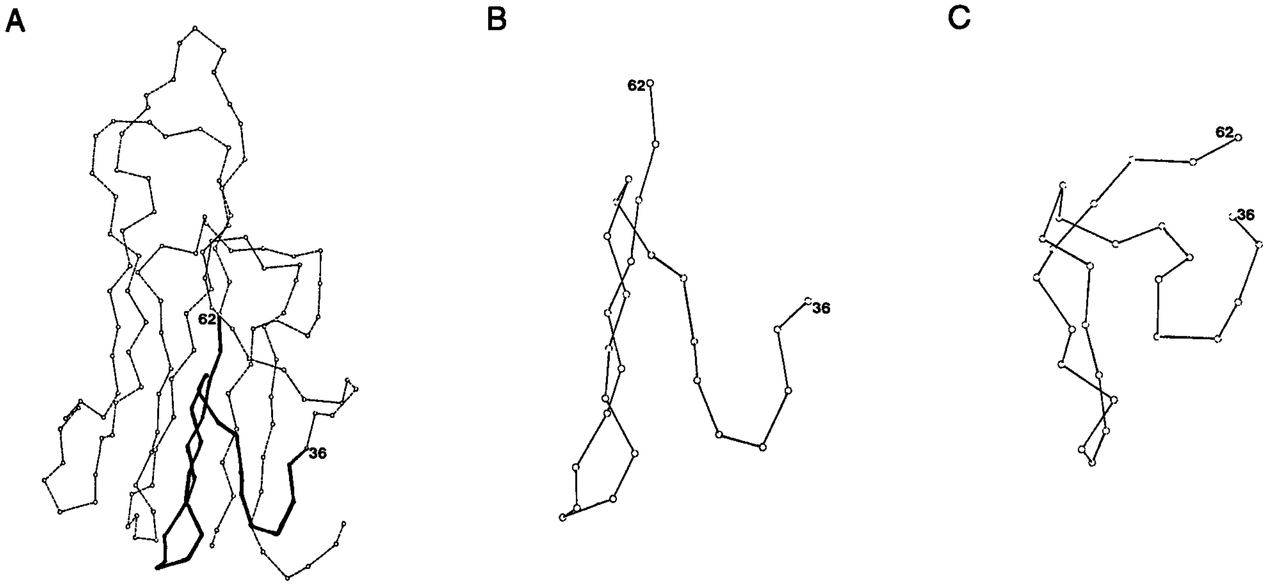

FIG. 1. Drawing of the TNFα monomer and the TNFα 36–62 peptide. (A) Drawing of the α-carbon backbone of the minimized crystal structure of TNFα. The sequence consisting of residues 36–62 are blackened. (B) Drawing of the α-carbon backbone of the TNFα sequence 36–62. (C) Drawing of the minimized structure of the TNFα 36–62 α-carbon backbone based on molecular modelling, using Computer Graphics.

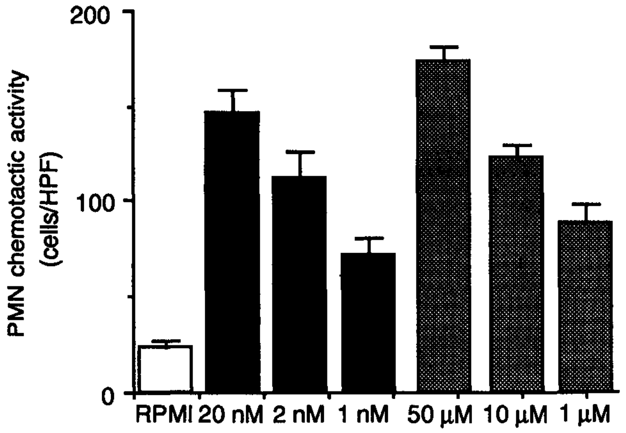

FIG. 2. Dose dependent effect of TNFα and TNFα 36–62 on PMN migration. Indicated concentrations of TNFα (■), and TNFα 36–62 (■) were tested for chemotactic effect on PMN with RPMI (□) as a control. Migrated cells were counted in high-power fields (HPF) (× 60 objective). Results are presented as means ± S.E.M. ($n = 9$).

**Table 1.** Effect of varying concentrations of TNFα 36–62 peptide on PMN migration.

| TNFα 36–62 concentration in lower compartment | TNFα 36–62 concentration in upper compartment | | | |
|---|---|---|---|---|
| | 0 | 1 μM | 10 μM | 50 μM |
| 1 μM | 91 ± 11 | 63 ± 13* | 54 ± 13 | 61 ± 11 |
| 10 μM | 129 ± 10 | 89 ± 7 | 64 ± 8* | 57 ± 14 |
| 50 μM | 197 ± 9 | 161 ± 16 | 111 ± 8 | 51 ± 9* |

The indicated concentrations of TNFα 36–62 peptide or medium alone were added to the upper compartments of the chemotaxis chamber, to neutralize the chemotactic effect of TNFα 36–62 used in the lower compartments. Migrated PMN were counted in high-power fields (HPF) (× 60 objective). The data represent the mean ± S.E.M. ($n = 9$). *The number of migrating PMN with the peptide present at the same concentration in both compartments.

tration of TNFα 36–62 was needed to induce migration of the same number of cells as induced by TNFα, is probably due to lack of domains necessary for optimal binding of TNFα 36–62.[12] FMLP was used as a positive control at $10^{-7}$ M, and gave an 11-fold migration of PMN compared to the negative control RPMI. As for TNFα, a Zigmond–Hirsch checkerboard analysis confirmed that TNFα 36–62 displayed chemotactic, and not chemokinetic effects on PMN (Table 1). A different TNFα peptide including residues 78–96, used at the same concentrations as TNFα 36–62, did not exhibit any chemotactic effect on PMN. We also tested whether TNFα and TNFα 36–62 had chemotactic effects on PBMC, and observed that both stimulants induced chemotatic activities on PBMC in a dose dependent manner and at similar concentrations as for PMN (data not shown). Both stimulants with their buffers were tested and confirmed free of LPS using the Endospecy assay.

*Inhibition of TNFα and TNFα 36–62-induced chemotaxis on PMN:* Both mAb, Utr-1 and Htr-7b, specific for p75 and p55 respectively, significantly inhibited the chemotactic response of TNFα on PMN (Fig. 3A), when used separately or in combination. Likewise, the chemotactic effect of TNFα 36–62 peptide was also significantly inhibited by Utr-1 or Htr-7b (Fig. 3B) or a combination of both mAb. These mAb alone, or in combination, did not induce chemotaxis on PMN. FMLP induced migration was not inhibited by these mAb alone or in combination. The experiments were repeated four times, and similar results were obtained despite donor variations. Antibodies against IL-8 or MCP-1 did not show any inhibitory effect on either the TNFα, TNFα 36–62 or

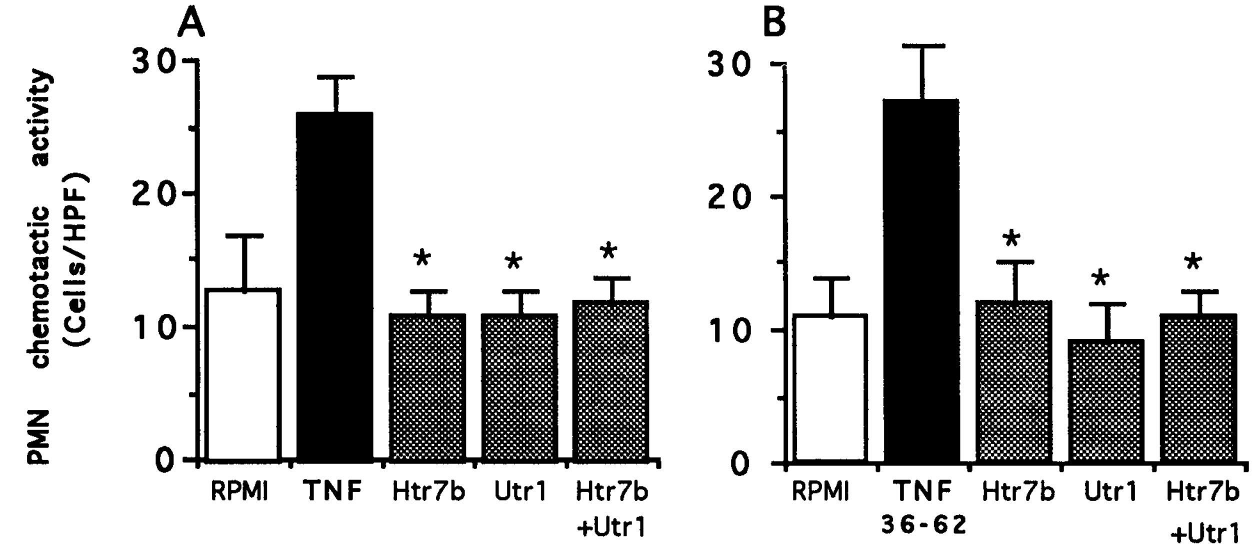

FIG. 3. Inhibitory effect of anti-p55 and anti-p75 antibodies on the chemotactic effect on PMN, induced by TNFα and TNFα 36–62. Utr-1(10 mg/ml) and Htr-7b(10 mg/ml) (anti-p75 and anti-p55, respectively) were preincubated with cells for 10 min at 4°C. the PMN were then tested for chemotaxis toward (A) 0.6 nM TNFα and (B) 10 μM TNFα 36–62 with (▩) or without (■) antibodies (□:control). Migrated cells were counted in high-power fields (HPF) (× 100 objective). The data represent the mean ± S.E.M. ($n = 8$). $^*p < 0.001$ compared to TNFα or TNFα 36–62 induced chemotactic effect without antibodies.

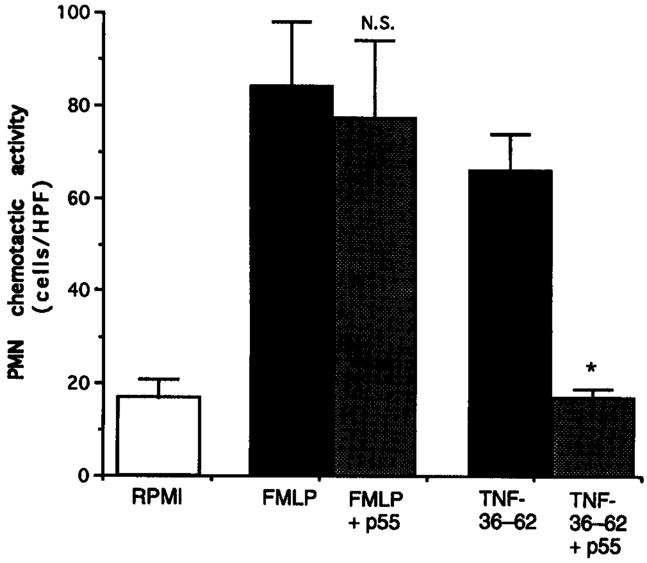

FIG. 4. Inhibitory effect of the soluble p55 receptor on PMN chemotaxis induced by TNFα 36–62. TNFα 36–62 or FMLP were tested for their ability to induce migration of PMN with (▩) or without (■) soluble p55 (□: control). Soluble p55 was preincubated with TNFα 36–62 or FMLP for 10 min at 20°C. Migrated cells were counted in high-power fields (HPF) (×100 objective). The results are expressed as mean ± S.E.M. ($n = 6$). $^*p < 0.001$ compared with TNFα 36–62 alone. N.S. = not significant.

FMLP induced chemotaxis on PMN when used in the same concentrations as Utr-1 and Htr-7b.

*TNFα 36–62 interacted with soluble p55:* The soluble p55 was able to significantly inhibit the chemotactic response of the TNFα 36–62 peptide on PMN (Fig. 4). Soluble p55 did not inhibit the chemotactic effect of FMLP (Fig. 4), indicating a specific binding of TNFα 36–62.

*TNFα 36–62 had no cytotoxic effect:* TNFα 36–62 was tested for cytotoxic activity. In contrast to TNFα, TNFα 36–62 did not show any cytotoxic effect on the WEHI 164 clone 13 fibrosarcoma cell line, when tested up to a $10^4$-fold higher concentration than TNFα.

### Discussion

In the present study it is shown that both TNFα 36–62 and TNFα is a chemoattractant to PMN *in vitro*. It is also shown that the chemotaxis induced by TNFα and TNFα 36–62 is mediated through both p55 and p75 TNF receptors. The finding regarding TNFα as a chemoattractant is in line with previous reports showing that antibodies against TNFα inhibit TNFα induced chemotaxis *in vitro* on PMN[3] and monocytes,[5] respectively. It is noticeable that there have been conflicting reports about the chemotactic property of TNFα.[2–5,23,24] In the authors' opinion the discrepancy might be explained by variations in experimental conditions, donor variations and sensitivities of the test systems. TNFα has also been shown to stimulate production of the chemoattractant IL-8 by granulocytes and monocytes.[25] Since interleukin-8 (IL-8) shows chemotactic activity for PMN,[20,26] TNFα might induce release of IL-8 by PMN, which in turn could be responsible for the chemotactic effect observed in our experiment. However, the present work shows that anti-IL-8 and anti-MCP-1 antibodies did not inhibit the chemotactic response of TNFα. This excludes IL-8 and MCP-1 as responsible for the chemotactic activity observed.

It is shown that the chemotactic activity TNFα on PMN involves both p55 and p75 receptors. Even non-redundancy in the function of the two receptors for some effects has been observed,[27] a simultaneous involvement of both p55 and p75 receptors has been reported for a series of TNFα activities, such as induction of differentiation of ML-1 cells, NF-κB activation, cytotoxicity on U937 cells and IL-6 production by endothelial cells.[27–29] Tartaglia *et al.*[30] have suggested that the high affinity p75 receptor may regulate the rate of TNFα association with the p55 receptor, by increasing the local concentration of TNFα through rapid ligand association and dissociation. In contrast to this, Brouckaert *et al.*[31] have proposed an alternative cooperation between the two receptors where p75 interferes with the p55 signalling pathway. This hypothesis is based on the fact that p75 triggering is not sufficient to initiate the redundant signals and that p75 triggering can diminish p55 mediated c-fos induction. However, it remains to be explored how the two receptors cooperate in TNFα induced chemotaxis.

Similar to TNFα, the TNFα 36–62 peptide also induced chemotaxis on PMN and PBMC. The finding that soluble p55 inhibited this effect (Fig. 3), indicates that the peptide possesses the conformation needed for interaction with the receptor. This supports the results of the molecular dynamic calculations (Fig. 1). That TNFα 36–62 is able to interact with soluble p55, is also in line with the recent work by Ratjen *et al.*[13] who found that several bioactive TNFα peptides were able to inhibit binding of TNFα to the TNF receptors. Antibodies against p55 and p75 inhibited TNFα 36–62 induced chemotaxis on PMN, which indicates that this peptide, like TNFα, induces the chemotactic activity through both TNF receptors. It is noticeable that Postlewaite *et al.*[12] have observed chemotaxis on fibroblasts, by using another TNFα peptide, which also included the sequence 36–62. Although these authors did not show that the chemotaxis induced by the peptide was TNF receptor mediated, desensitisation studies suggested involvement of TNF receptors. The present work supports and extends this hypothesis.

TNFα 36–62 was not cytotoxic, suggesting the existence of distinct TNFα regions for the cytotoxic and chemotactic effects. It is noteworthy that two other TNFα peptides, which overlap with only four residues (54–58), were shown to be cytotoxic,[13] suggesting a critical domain for TNFα cytotoxicity. This sequence is included in our TNFα 36–62 peptide and the molecular calculations of the peptide suggested that this specific domain encompassing residues 54–58 did not possess the conformation needed for optimal interaction with the TNF receptors. This was due to the attraction between the oppositely charged C- and N-terminals (Fig. 1).

Crystallographic studies on the TNFβ/TNF receptor complex have shown that three TNFβ monomers bind three TNF receptors in a symmetrical fashion.[32] It has been suggested that a crosslinking of TNF receptor is also necessary for signal transduction leading to TNFα effects,[33–35] and is based on studies with antibodies and TNFα mutants against TNF receptors.[33–36] Our and other investigations with synthetic TNFα peptide fragments,[12–14] however, suggest that at least some of the TNFα effects are not dependent on crossreaction of TNF receptors with three TNFα monomers.

In conclusion, different TNFα peptides may induce distinct activities, indicating that TNFα possess distinct domains critical for different TNFα activities. This property opens the possibility of designing TNFα fragments with specific TNFα effects. We are currently investigating this hypothesis by studying TNFα 36–62 and several other TNF peptides for their bioactivities and specificities to target cells.

### References

1. Yoshimura T, Leonard EJ. Human monocyte chemoattractant protein-1 (MCP-1). In: Westwick J, Lindley IJD, Kunkel SL, eds. *Advances in Experimental Medicine and Biology. Chemotactic cytokines.* New York: Plenum Press. 1991; **305:** 47–53.
2. Ming WJ, Bersani L, Mantovani A. Tumor necrosis factor is chemotactic for monocytes and polymorphonuclear leukocytes. *J Immunol* 1987; **138:** 1469–1474.
3. Newman I, Wilkinson PC. Chemotactic activity of lymphotoxin and tumour necrosis factor alpha for human neutrophils. *Immunology* 1989; **66:** 318–320.
4. Figari I, Mori NA, Palladino MA. Regulation of neutrophil migration and superoxid production by recombinant tumor necrosis factor-α and -β: comparison to recombinant interferon-γ and interleukin-1α. *Blood* 1987; **70:** 979–984.
5. Wang MJ, Walter S, Mantovani A. Reevaluation of the chemotactic activity of tumor necrosis factor for monocytes. *Immunology* 1990; **71:** 364–367.
6. Zhang Y, Ramos BF, Jakchik BA. Neutrophil recruitment by tumor necrosis factor from mast cells in immune complex peritonitis. *Science* 1992; **258:** 1957–1959.
7. Issekutz AC, Issekutz TB. Quantitation and kinetics of blood monocyte migration to acute inflammatory reactions, and Il-1α, tumor necrosis factor-α and IFN-γ. *J Immunol* 1993; **151:** 2105–2115.
8. Goeddel DV, Aggarwall BB, Gray PW, *et al.* Tumor necrosis factors: gene structure and biological activities. *Cold Spring Harbor Symp Quant Biol* 1986; **51:** 597–609.
9. Beutler B, Cerami A. Tumor necrosis, cachexia, shock, and inflammation: A common mediator. *Ann Rev Biochem* 1988; **57:** 505–518.
10. Vilcek J, Lee TH. Tumor necrosis factor. New insights into the molecular mechanisms of its multiple actions. *J Biol Chem* 1991; **266:** 7313–7316.
11. Halvorsen H, Olsen JO, Østerud B. Granulocytes enhance LPS-induced tissue factor activity in monocytes via an interaction with platelets. *J Leukoc Biol* 1993; **54:** 275–282.
12. Postletwaite AE, Seyer JM. Stimulation of fibroblast chemotaxis by human recombinant tumor necrosis factor α (TNF-α) an a synthetic TNF-α peptide. *J Exp Med* 1990; **172:** 1749–1756.
13. Rathjen DA, Ferrante A, Aston R. Differential effects of small tumour necrosis factor peptides on tumour cell cytotoxicity, neutrophil activation and endothelial cell procoagulant activity. *Immunology* 1993; **80:** 293–299.
14. Kapas L, Hong L, Cady AB, *et al.* Somnogenic, pyrogenic, and anorectic activities of tumor necrosis factor-α and TNF-α fragments. *Am J Physiol* 1992; **263:** R708–R715.
15. Glen RC, Recent progress in computational chemistry and molecular graphics applied to drug design. *Drug News and Perspectives* 1990; **3:** 332–336.
16. Eck JE, Sprang RS. The structure of tumour necrosis factor-α at 2.6 Å resolution. *J Biol Chem* 1989; **264:** 17595–17605.
17. Bartnes K, Rekdal Ø, Briand JP, Hannestad K. Th1 clones that suppress IgG2a[b] specifically recognize an allopeptide determinant comprising residues 435–451 of γ2a[b]. *Eur J Immunol* 1993; **23** 2655–2660.
18. Brockhaus M, Schoenfeld HJ, Schlaeger EJ, Hunziker W, Lesslauer W, Loetscher H. Identification of two types of tumor necrosis factor receptors on human cell lines by monoclonal antibodies. *Proc Natl Acad Sci USA* 1990; **87:** 3127–3131.
19. Bøyum A. Isolation of mononuclear cells and granulocytes from human blood. *Scand J Clin Lab Invest* 1968; **21 (suppl):** 77–89.
20. Helset E, Sildnes T, Konopski ZS. Endothelin stimulates monocytes *in vitro* to release chemotactic activity identified as interleukin-8 and monocyte chemotactic protein-1. *Mediators of Inflammation* 1994; **3:** 155–160.
21. Espevik T, Nissen-Meyer J. A highly sensitive cell line, WEHI-164 clone 13, for measuring cytotoxic factor/tumor necrosis factor from human monocytes. *J Immunol Methods* 1986; **95:** 99–105.
22. Mosmann TR, Cherwinski H, Bond MW, Giedlin MA, Coffman RL. Two types of murine helper T cell clone I. Definition according to profiles of lymphokine activation and secreted proteins. *J Immunol* 1986; **137:** 2348–2357.
23. Kownatzki E, Kapp A, Uhrich S. Modulation of human neutrophilic granulocyte functions by recombinant human tumor necrosis factor and recombinant human lymphotoxin. Promotion of adherance, inhibition of chemotactic migration and superoxide anion release from adherent cells. *Clin Exp Immunol* 1988; **74:** 143–148.

24. Mrowietz U, Schröder JM, Christophers E. Recombinant human tumor necrosis factor-α lacks chemotactic activity for human peripheral blood neutrophils and monocytes. *Biochem Biophys Res Commun* 1988; **153:** 1223–1228.
25. Van Damme J. Granulocyte and monocyte chemotactic factors: stimuli and producer cells. In: Westwick J, Lindley IJD, Kunkel SL, eds. *Advances in Experimental Medicine and Biology. Chemotactic cytokines.* New York: Plenum Press. 1991; **305:** 1–9.
26. Baglioni M, Walz AO, Kunkel SL. Neutrophil-activating peptide/interleukin 8, a novel cytokine that activates neutrophils. *J Clin Invest* 1989; **84:** 1045–1049.
27. Tartaglia LA, Goeddel DW. Two TNF receptors. *Immunology Today* 1992; **13:** 151–153.
28. Takeda K, Iwamoto S, Takeda M. Roles of two tumor necrosis factor receptors in induction of differentiation of ML-1 cells. *Anticancer Research* 1993; **13:** 883–886.
29. Shalaby MR, Sundan A, Loetscher H, Brockhaus M, Lesslauer W, Espevik T. Binding and regulation of cellular functions by monoclonal antibodies against human tumor necrosis factor receptors. *J Exp Med* 1990; **172:** 1517–1520.
30. Tartaglia LA, Pennica D, Goeddel DV. Ligand passing: The 75-kDa tumor necrosis factor (TNF) receptor recruits TNF for signalling by the 55-kDa TNF receptor. *J Biol Chem* 1993; **268:** 18542–18532.
31. Brouckaert P, Libert C, Everaerdt B, Takahashi N, Cauwles A, Fiers W. Tumor necrosis factor, its receptors and the connection with interleukin 1 and interleukin 6. *Immunobiol* 1993; **187:** 317–329.
32. Banner DW, D'Àrcy A, Janes W, *et al.* Crystal structure of the soluble human 55 kDA TNF receptor–human TNF-β complex: Implication for TNF receptor activation. *Cell* 1993; **73:** 431–445.
33. Zhang XM, Weber I, Chen MJ. Site-directed mutational analysis of human tumor necrosis factor-α receptor binding site and structure–functional relationship. *J Biol Immunol* 1992; **267:** 24069–24075.
34. Engelmann H, Holtmann H, Brakebusch C, *et al.* Antibodies to a soluble form of a tumor necrosis factor (TNF) receptor have TNF-like activity. *J Biol Chem* 1990; **265:** 14497–14504.
35. Ostade XV, Tavernier J, Prange T, Fiers W. Localization of the active site of human tumor necrosis factor by mutational analysis. *The EMBO Journal* 1991; **10:** 827–836.
36. Espevik T, Brockhaus M, Loetscher H, Nonstad U, Shalaby R. Characterization of binding and biological effects of monoclonal antibodies against a human tumor necrosis factor receptor. *J Exp Med* 1990; **171:** 415–426.

ACKNOWLEDGEMENTS. We thank Eli Berg and Liv Tone Elliassen for excellent technical assistance. The contribution of Arnfinn Kvarsnes in performing the FAB-MS analysis of the synthetic peptides is gratefully acknowledged. The cost of the MS instrument is partially paid by the Norwegian Council for Science and the Humanities (NAVF), and the Norwegian Council for Agriculture Research (NLVF).

**Received 29 March 1994; accepted in revised form 25 May 1994**
